# κ-opioid receptor stimulation alleviates rat vascular smooth muscle cell calcification via PFKFB3-lactate signaling

**DOI:** 10.18632/aging.203050

**Published:** 2021-05-20

**Authors:** Jin Niu, Chen Wu, Min Zhang, Zhen Yang, Zhenhua Liu, Feng Fu, Jun Li, Na Feng, Xiaoming Gu, Shumiao Zhang, Yali Liu, Rong Fan, Juan Li, Jianming Pei

**Affiliations:** 1 Department of Physiology and Pathophysiology, National Key Discipline of Cell Biology, Fourth Military Medical University, Xi’an 710032, Shaanxi Province, China; 2 Department of Healthcare of 940 Hospital, Joint Logistics Support Force of PLA, Lanzhou 730000, Gansu Province, China; 3 Department of Neurology, Xinjiang Military General Hospital, Urumqi 830000, Xinjiang Province, China; 4 Department of College of Life Sciences, Northwest University, Xi’an 710032, Shaanxi Province, China

**Keywords:** κ-opioid receptor, vascular calcification, vascular smooth muscle cells, PFKFB3, lactate

## Abstract

In the present study, the effects and mechanism of action of U50,488H (a selective κ-opioid receptor agonist) on calcification of rat vascular smooth muscle cells (VSMCs) induced by β-glycerophosphate (β-GP) were investigated. VSMCs were isolated and cultured in traditional FBS-based media. A calcification model was established in VSMCs under hyperphosphatemia and intracellular calcium contents. Alkaline phosphatase (ALP), lactate dehydrogenase (LDH), and lactate were detected in cell culture supernatants before and after treatment. Alizarin red staining was used to detect the degree of calcification of VSMCs. Expression levels of key molecules of osteogenic markers, fructose-2,6-biphosphatase 3 (PFKFB3), and proline hydroxylase 2 (PHD2), were determined using western blotting. Further, vascular calcification was induced by vitamin *D3* plus nicotine in rats and isolated thoracic aortas, calcium concentration was assessed in rat aortic rings *in vitro*. We demonstrated that U50,488H inhibited VSMC calcification in a concentration-dependent manner. Moreover, U50,488H significantly inhibited osteogenic differentiation and ALP activity in VSMCs pretreated with β-GP. Further studies confirmed that PFKFB3 expression, LDH level, and lactate content significantly increased during calcification of VSMCs; U50,488H reversed these changes. PHD2 expression showed the opposite trend compared to PFKFB3 expression. nor-BNI or 3-PO abolished U50,488H protective effects. Besides, U50,488H inhibited VSMC calcification in rat aortic rings *ex vivo*. Collectively, our experiments show that κ-opioid receptor activation inhibits VSMC calcification by reducing PFKFB3 expression and lactate content, providing a potential drug target and strategy for the clinical treatment of vascular calcification.

## INTRODUCTION

Vascular calcification (VC) is an active pathophysiological process in which mesenchymal cells, especially vascular smooth muscle cells (VSMCs), transdifferentiated into an osteo-/chondroblast-like phenotype under the action of complex factors, resulting in local abnormal calcium precipitation and vascular tissue mineralization [1, 2]. VC reduces vascular compliance, causes local hypoxia, and increases the risk of vascular rupture and aneurysm formation, which is related to atherosclerosis and comorbidities, such as diabetes, heart failure, and chronic kidney disease (CKD) [3]. Although this process is traditionally considered passive, degenerative, and quiescent, recent studies suggested that VC is an active process beginning with the deposition of cell-derived matrix vesicles into the extracellular matrix, where osteogenic differentiation plays a predominant role [4–6]. Ectopic osteogenesis driven by oxidative stress and metabolic are responsible for VC initiation [7, 8].

Glycolysis is related to the osteogenic transformation and the VSMC phenotype switch [9–11]. PFKFB3 is a key enzyme in glycolysis [12]. Previous studies have confirmed that PFKFB3 is related to diabetic atherosclerosis and vascular remodeling [13]. Lactate is the end product of anaerobic glycolysis [14]. The glycolysis pathway is reportedly activated under the action of a variety of calcification factors and produces excessive glycolysis products [15]. Observations in hypoxia-cultured VSMCs were like those in lactate-cultured VSMCs [16]. A recent VSMC calcification study revealed that lactate accelerates osteoblastic phenotype transition in VSMCs through BNIP3-mediated mitophagy suppression [17]. They also investigated the specific links between lactate and VC. Mechanistically, lactate enhanced fission but halted mitophagy via activation of the NR4A1/DNA-PKcs/p53 pathway, finally accelerating calcium deposition and osteoblastic phenotype transition in VSMCs [18]. Therefore, glucose metabolism is closely associated with VC [18, 19]. Approximately 70.1% of total ATP originated from glycolysis in human aortic VSMCs under normal conditions, and β-glycerophosphate (β-GP)-induced osteo-/chondrogenic transdifferentiation and calcification of VSMCs were proven to be more oxidative and less glycolytic, which indicates that interference of VSMC metabolic pathways may regulate VC progression [9, 20].

Of note, the cell’s major energetic source is glycolysis, which is stimulated by hypoxia, and VC is related to hypoxia. Hypoxia is a primary condition contributing *to* VC [21]. Hypoxic gene activation is linked to CKD and stimulates bone cell osteogenic differentiation [22]. Although a direct link between hypoxia and VC has not been described, studies have associated hypoxia-inducible factor-1 (HIF-1) with VC [23]. Therefore, hypoxic signaling may indeed be implicated in VSMC osteogenic transdifferentiation, leading to VC [24]. The human genome encodes three potential HIF-1a prolyl-4-hydroxylases, designated prolyl hydroxylase domain-containing proteins 1, 2, and 3 (PHD1, PHD2, and PHD3), each of which is capable of hydroxylating two proline residues within a HIF-1a peptide substrate *in vitro* [25]. Phd2 is most abundant at the protein level in most organs [26]. HIF-1a activation leads to increased PFKFB3 expression in type 1 diabetes, leading to increased glycolysis products such as lactic acid and LDH [13]. Previous studies have confirmed that PHD2 inhibited VSMC proliferation by regulating HIF-1a expression, but it is unknown whether it can regulate PFKFB3 level in VC [27].

The κ-opioid receptor (κ-OR) is predominantly expressed in the cardiovascular system [28]. As a selective agonist of κ-OR, U50,488H can improve hemodynamics and vascular endothelial function and reverse vascular remodeling by activating cardiovascular κ-OR [28–30]. Previous studies have confirmed that a higher dose of U50,488H significantly prevented the myocardial ischemia and reperfusion (I/R) injury-induced increase in myocardial lipid peroxidation and depletion of myocardial antioxidants and that κ-OR stimulation exerts a protective effect on vascular endothelium [31, 32]. However, the impact of κ-OR stimulation on vascular remodeling during VC remains elusive. Details regarding the osteo-/chondrogenic transdifferentiation of VSMCs and the underlying mechanism are largely unknown [33]. At present, whether κ-OR stimulation reverses the osteogenic transformation of VSMCs, improves the local calcium-phosphorus ratio, and glycolysis level has not yet been reported.

In this study, the effect of U50,488H on VSMC exposed to β-GP, parameters of calcification degree, and calcification-related proteins were examined. The influence of U50,488H and nor-BNI on key modulators of glycolysis, namely fructose-2,6-biphosphatase 3 (PFKFB3), PHD2, LDH, and lactate content, were observed. We also attempted to explore the role of κ-OR stimulation in a rat VC model and further analyzed the effect of U50,488H on the calcification level of rat thoracic aorta cultured *in vitro*. Thus, in this study, we sought to provide an experimental and theoretical basis to further understand VC pathogenesis and prevention.

## RESULTS

### Effect of κ-OR stimulation on VSMC calcification induced by β-GP

**Figure 1:**
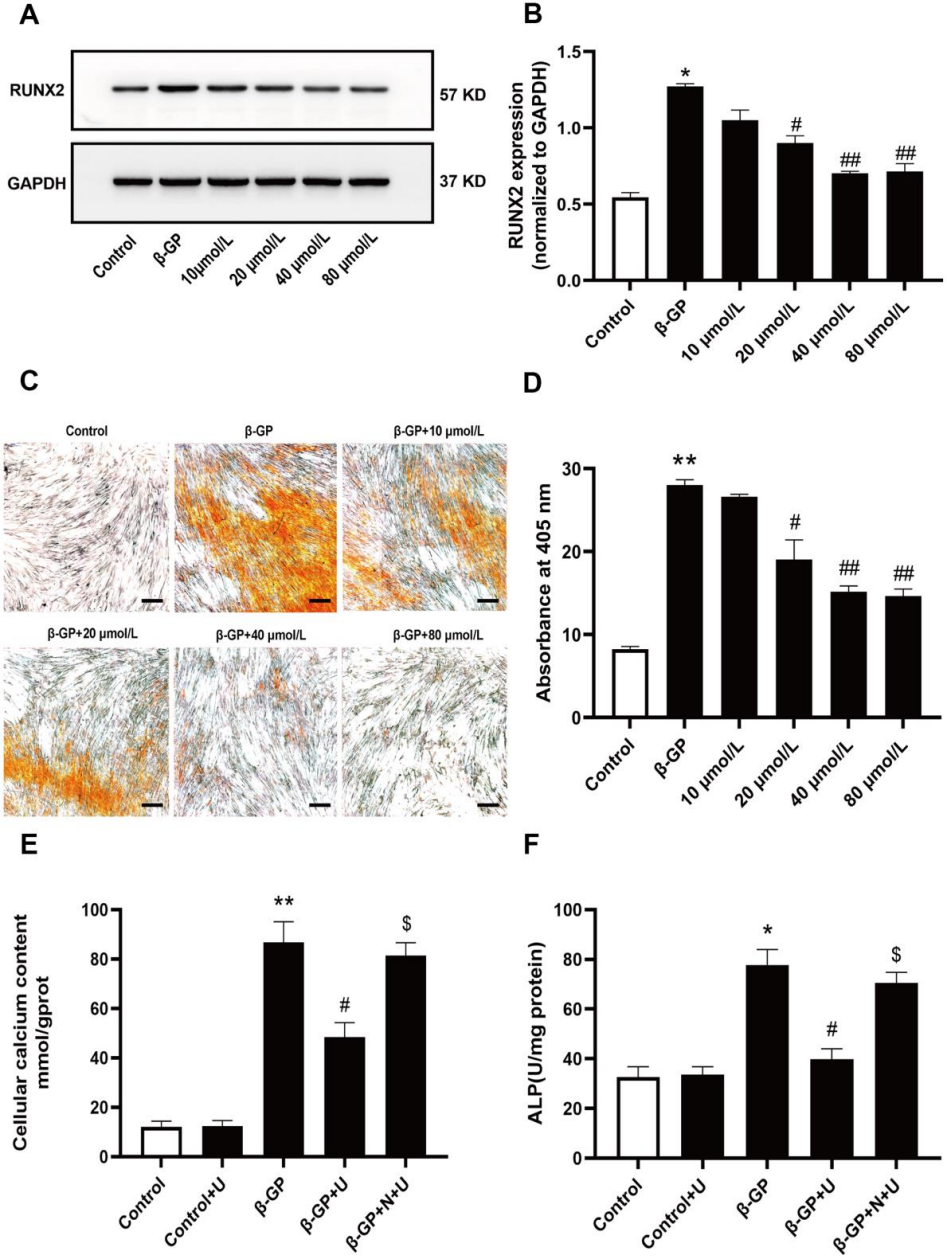
**Effects of κ-OR stimulation on VSMC calcification induced by β-GP.** Control: VSMCs were incubated in the absence of β-GP. β-GP: VSMCs were incubated in 10% FBS-DMEM containing β-GP (10 mmol/L) for ten days. U50,488H (10 - 80 mmol/L) was added before β-GP treatment to investigate the effect of U50,488H on VSMC calcification. (**A**) Cell lysates were collected and analyzed for RUNX2 using western blotting. (**B**) Quantitative analysis of RUNX2 expression. (**C**) Calcium nodules were stained with Alizarin red. Red nodules indicate calcium deposition. Scale bar = 50 μm. (**D**) Quantification of mineralization. (**E**, **F**) The contents of intracellular calcium and ALP were detected using calcium assay kits and ALP activity kits. U, U50,488H; β-GP, β-Glycerophosphate disodium salt pentahydrate; N, nor-BNI; Data obtained from quantitative densitometry were presented as means ± SEM. n=4 in each group. ^*^P < 0.05 versus the control group, ^**^P < 0.01 versus the control group, ^#^*P* < 0.05 versus the β-GP group, ^##^*P* < 0.01 versus the β-GP group, ^$^*P* < 0.05 versus the β-GP+U group.

To determine the effect of κ-OR stimulation on VSMC calcification induced by high phosphorus, VSMCs were treated with β-GP in the presence or absence of various concentrations of U50,488H (10–80 μmol/L) for ten days. After ten days of treatment, the VSMC calcification cell model was successfully established, confirming that U50,488H dose-dependently inhibited the expression of osteogenic specific transcription factor (RUNX2), of which 40 μmol/L U50,488H elicited the most evident inhibitory effect on osteogenic differentiation of VSMCs (the decrease was up to approximately 60.94% in 40 μmol/L); therefore, this dose was used in all subsequent experiments (Figure 1A, 1B). An inhibitory effect of U50,488H on calcium deposition was also observed by Alizarin red staining. After U50,488H treatment, the amount of calcium deposition decreased to 56.42% (Figure 1C, 1D). The intracellular calcium and ALP contents in VSMCs increased significantly with 10-day β-GP treatment (619.32% and 152.12% compared with the control group) and were markedly attenuated by U50,488H, the decrease of intracellular calcium was 34.18 %, and ALP content was 48.62 % (*P* < 0.05). This effect of U50,488H was abolished by nor-BNI treatment (*P* < 0.05) (Figure 1E, 1F). These results suggest that κ-OR stimulation significantly attenuates β-GP-induced calcification in VSMCs.

### Effects of κ-OR stimulation on the expression of osteogenic differentiation proteins in VSMCs treated with β-GP

**Figure 2:**
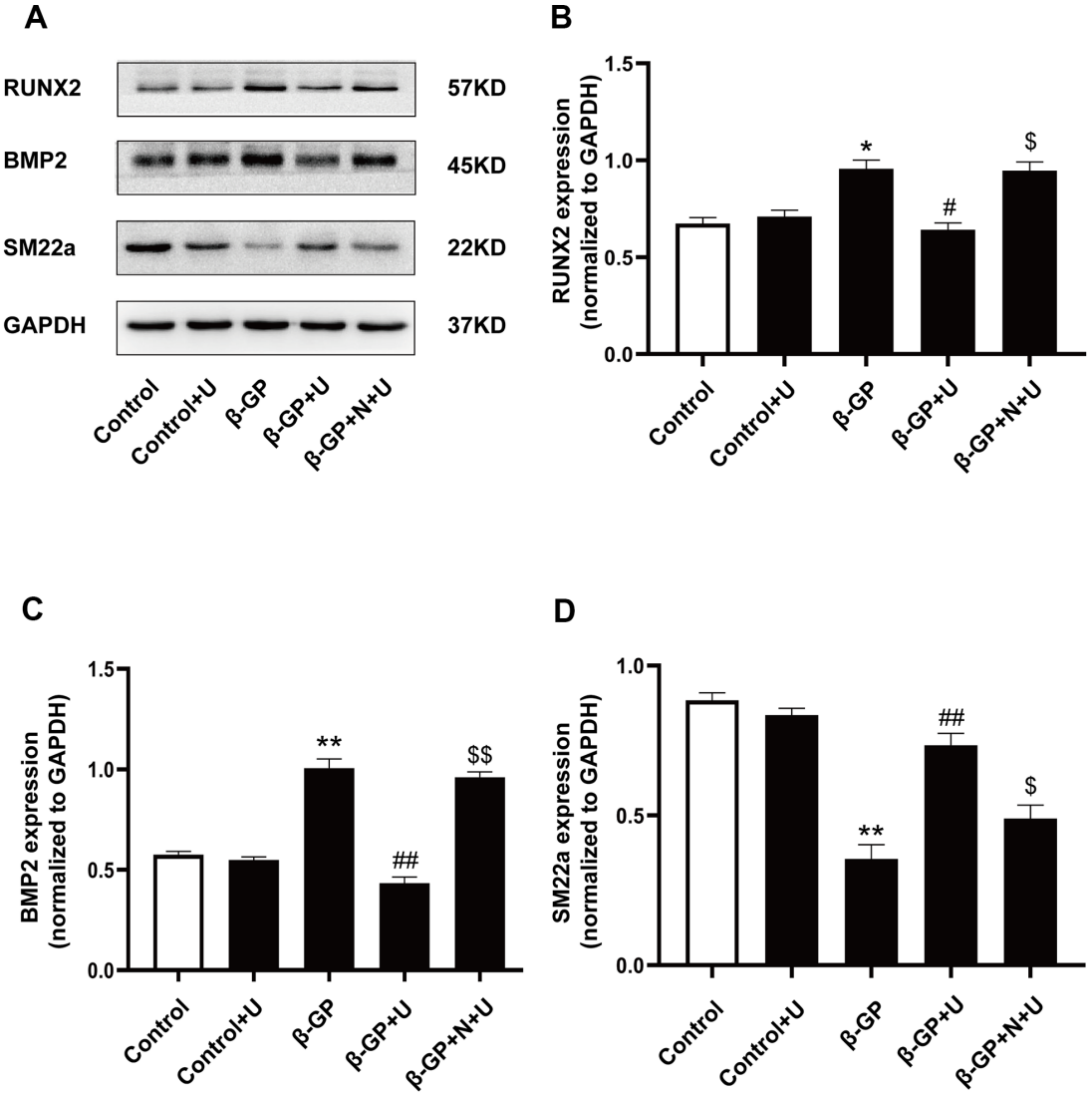
**Effects of κ-OR stimulation on osteogenic protein expression in VSMCs treated with β-GP.** (**A**) Representative blot images of osteogenic differentiation-associated proteins. (**B**) Quantitative analysis of RUN2 protein expression using densitometry. (**C**) Quantitative analysis of BMP2 protein expression using densitometry. (**D**) Quantitative analysis of SM22a protein expression using densitometry. U, U50,488H; β-GP, β-Glycerophosphate disodium salt pentahydrate; N, nor-BNI; Data obtained from quantitative densitometry were presented as means ± SEM. n=4 in each group. ^*^P < 0.05 versus the control group, ^**^P < 0.01 versus the control group, ^#^*P* < 0.05 versus the β-GP group, ^##^*P* < 0.01 versus the β-GP group, ^$^*P* < 0.05 versus the β-GP+U group, ^$$^*P* < 0.01 versus the β-GP+U group.

To further investigate the effect of κ-OR stimulation on osteogenic differentiation, we examined RUNX2, BMP2, and SM22a protein levels via western blotting *in vitro*. As shown in Figure 2A–2C, the expression of RUNX2 and BMP2 was significantly increased in the β-GP group compared with the control group (*P* < 0.05). After U50,488H treatment, RUNX2 and BMP2 expression was significantly decreased (*P* < 0.05). However, the inhibitory effect of U50,488H was abolished by nor-BNI treatment (*P* < 0.05). Besides, the expression of SM22a (a smooth muscle protein) was significantly decreased in the β-GP group (*P* < 0.01), which increased significantly in the U50,488H treatment group (*P* < 0.01), and these effects were significantly reduced by nor-BNI (*P* < 0.05) (Figure 2A, 2D). These data suggest that κ-OR stimulation inhibits osteogenic differentiation.

### Effects of κ-OR stimulation on the expression of PFKFB3, PHD2 and glycolysis products in VSMCs treated with β-GP

**Figure 3:**
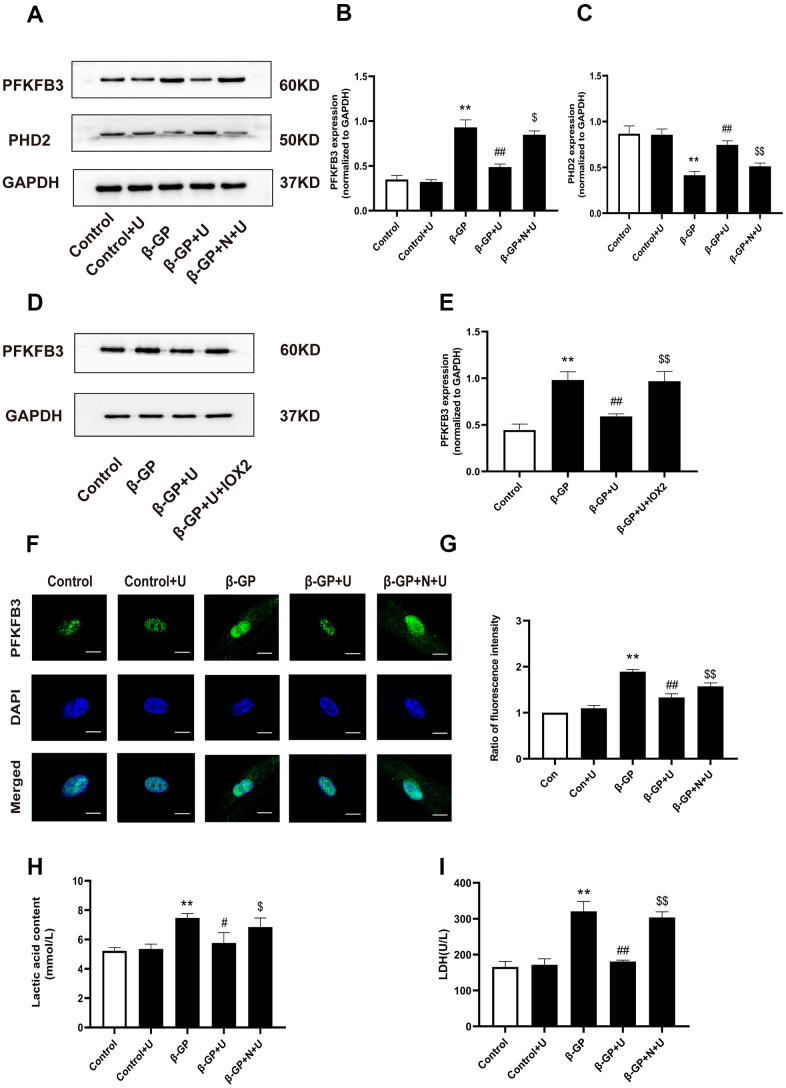
**Effects of κ-OR stimulation on the expression of PFKFB3, PHD2, and glycolysis products in VSMCs treated with β-GP.** (**A**) Representative blot images of PFKFB3 and PHD2. (**B**) Quantitative analysis of PFKFB3 protein expression using densitometry. (**C**) Quantitative analysis of PHD2 protein expression using densitometry. (**D**) Representative blot images of PFKFB3. (**E**) Quantitative analysis of PFKFB3 protein expression using densitometry. (**F**) After various treatments, PFKFB3 nuclear translocation was evaluated via immunofluorescence using confocal microscopy. At least 10-15 cells per condition were imaged. Scale bar = 10μm. (**G**) Quantification of PFKFB3 immunofluorescence intensity. (**H**, **I**) Lactic acid content and LDH levels were detected. U, U50,488H; β-GP, β-Glycerophosphate disodium salt pentahydrate; N, nor-BNI; Data obtained from quantitative densitometry were presented as means ± SEM. n=5 in each group. ^**^P < 0.01 versus the control group, ^#^*P* < 0.05 versus the β-GP group, ^##^*P* < 0.01 versus the β-GP group, ^$^*P* < 0.05 versus the β-GP+U group, ^$$^*P* < 0.01 versus the β-GP+U group.

We further investigated the mechanism by which κ-OR stimulation affects VSMC calcification and glycolysis. The expression of PFKFB3 in the β-GP group was significantly higher than that in the control group (*P* < 0.01), which was decreased by U50,488H (*P* < 0.01), whereas this effect was abolished by nor-BNI (*P* < 0.05) (Figure 3A, 3B). The expression of PHD2 in the β-GP group was significantly decreased relative to the control group (*P* < 0.01), in which it was significantly increased by U50,488H (*P* < 0.01). Of note, this effect was abolished by nor-BNI (*P* < 0.01) (Figure 3A, 3C), indicating that κ-OR stimulation reduced PFKFB3 expression and increased the expression of PHD2 during calcification of VSMCs treated with β-GP. To explore the association between PHD2 and PFKFB3, we used IOX2, a specific inhibitor of PHD2, to inhibit PHD2 and observe changes in PFKFB3. Results showed that U50,488H significantly inhibited PFKFB3 expression compared with the β-GP group, while IOX2 abolished this effect (*P* < 0.01, Figure 3D, 3E). To further clarify the association between κ-OR stimulation and expression of PFKFB3, normal or calcified VSMCs were incubated with or without U50,488H for ten days, and the cellular PFKFB3 distributions were visualized by immune fluorescence staining. The images indicated that U50,488H significantly inhibited PFKFB3 expression by 16.45% in the nuclei than the β-GP group, while this effect was abolished by nor-BNI (*P* < 0.05, Figure 3C, 3D). Besides, compared with the control group, lactic acid levels increased by up to 42.33% and LDH in the β-GP group increased 83.72% compared with the control group, and U50,488H treatment decreased 34.42% lactic acid content and 22.89% LDH activity and in VSMCs cells after ten days (*P* < 0.05). However, the inhibitory effect of U50,488H was abolished by nor-BNI treatment (*P* < 0.05, Figure 3E, 3F). These results reveal that κ-OR stimulation significantly decreases PFKFB3 expression and glycolysis production in VSMC calcification induced by β-GP.

### Inhibition of PFKFB3 expression reversed VSMC calcification induced by β-GP

**Figure 4:**
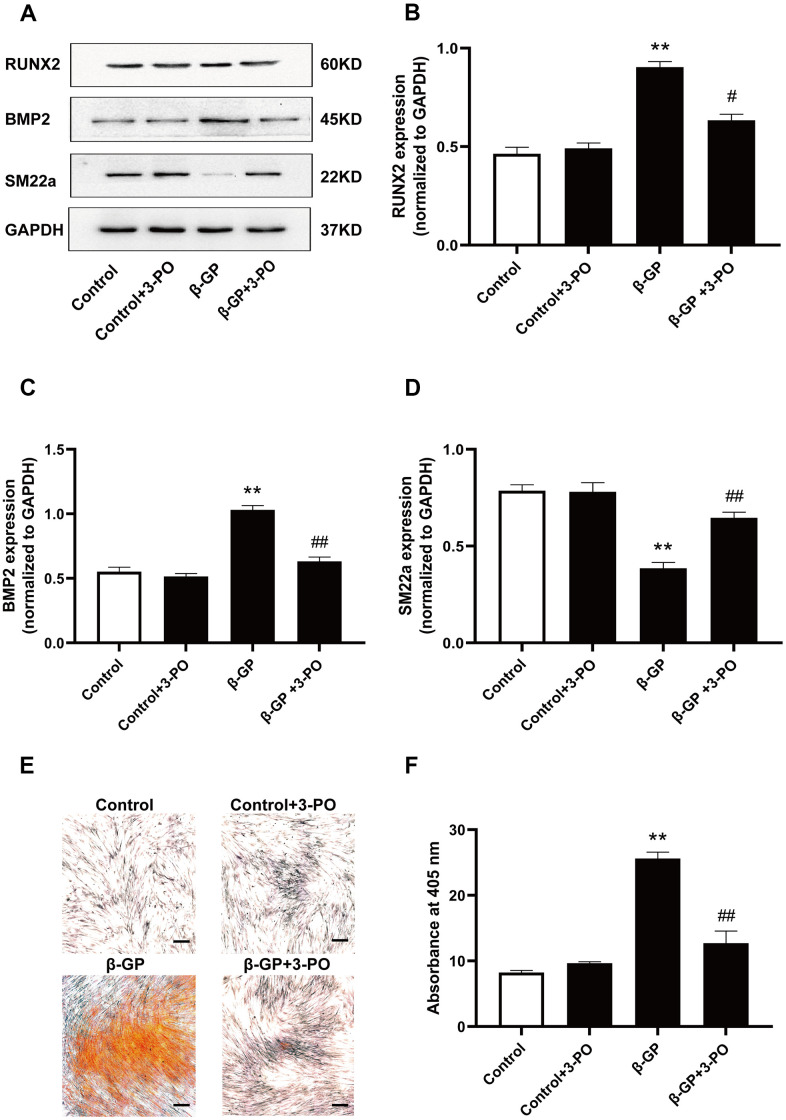
**Effects on VSMC calcification induced by inhibiting PFKFB3 expression by β-GP.** (**A**) Representative blot images of osteogenic differentiation-associated proteins. (**B**) Quantitative analysis of RUN2 protein expression using densitometry. (**C**) Quantitative analysis of BMP2 protein expression using densitometry. (**D**) Quantitative analysis of SM22a protein expression using densitometry. (**E**) Calcium nodules were stained with Alizarin red. Red nodules indicate calcium deposition. Scale bar = 50μm. (**F**) Quantification of mineralization. β-GP, β-Glycerophosphate disodium salt pentahydrate; Data were normalized using log_10_ and analyzed using one-way ANOVA tests; U, U50,488H; 3-PO, a novel small molecule inhibitor of the PFKFB3 isozyme; Data obtained from quantitative densitometry were presented as means ± SEM. n=5 in each group. ^**^P < 0.01 versus the control group, ^#^*P* < 0.05 versus the β-GP group, ^##^*P* < 0.01 versus the β-GP group.

**Figure 5:**
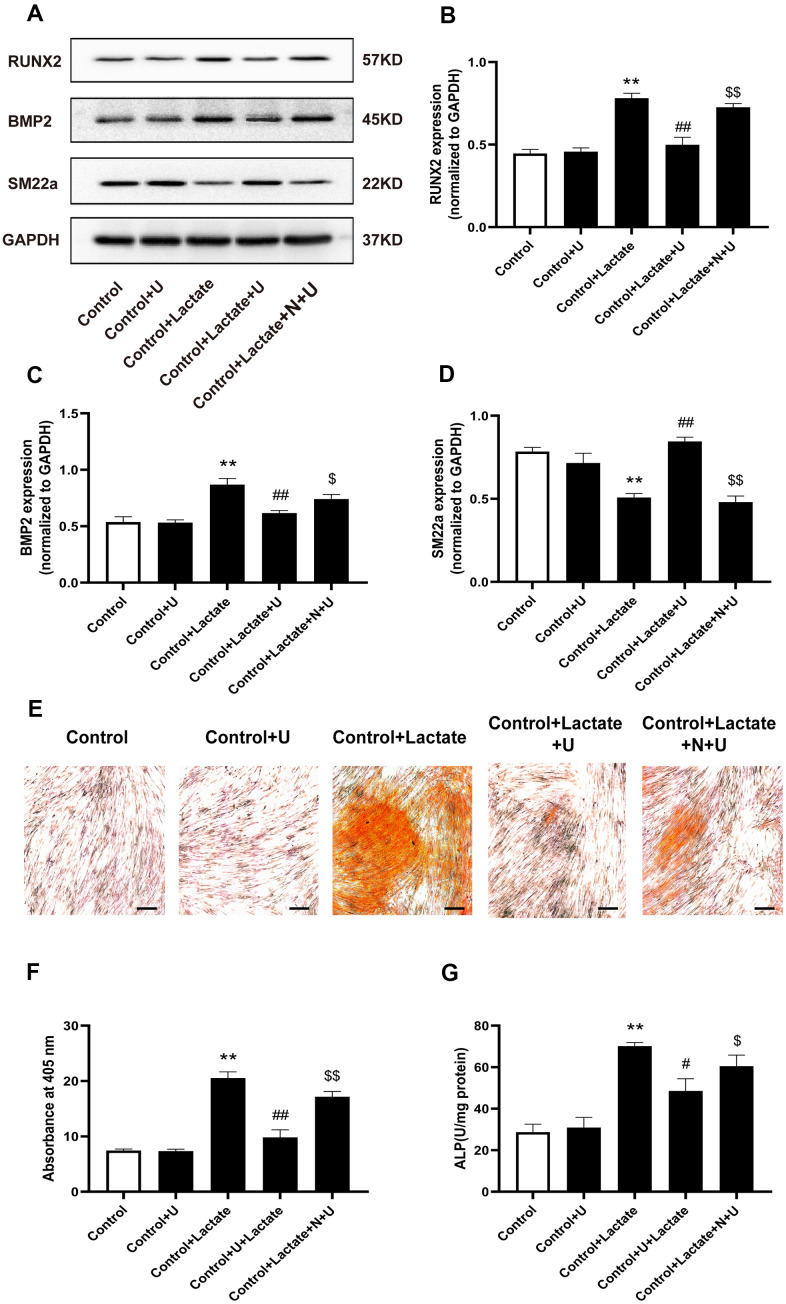
**Effects of κ-OR stimulation on the expression of osteogenic proteins in VSMCs treated with lactate.** (**A**) Representative blot images of osteogenic differentiation-associated proteins. (**B**) Quantitative analysis of RUN2 protein expression using densitometry. (**C**) Quantitative analysis of BMP2 protein expression using densitometry. (**D**) Quantitative analysis of SM22a protein expression using densitometry. (**E**) Calcium nodules were stained with Alizarin red. Red nodules indicate calcium deposition. Scale bar = 50 μm. (**F**) Quantification of mineralization (mean ± SEM; n = 6). (**G**) ALP activity was detected using ALP activity kits. Data were normalized using log_10_ and analyzed using one-way ANOVA tests; U, U50,488H; N, nor-BNI; Data obtained from quantitative densitometry were presented as means ± SEM. n=5 in each group. ^*^P < 0.05 versus the control group, ^**^P < 0.01 versus the control group, ^#^*P* < 0.05 versus the β-GP group, ^##^*P* < 0.01 versus the β-GP group, ^$^*P* < 0.05 versus the β-GP+U group, ^$$^*P* < 0.01 versus the β-GP+U group.

To explore the mechanism by which PFKFB3 affects VSMC calcification induced by β-GP, we attempted to use 3-PO, a small molecule inhibitor of PFKFB3, to inhibit PFKFB3 expression and observe changes in osteogenic differentiation proteins and calcified nodule formation. Results showed that RUNX2 and BMP2 expression was significantly increased by β-GP-induced calcification in VSMCs for ten days *(P* < 0.05, Figure 4A–4C), which was inhibited by treatment with 3-PO. In contrast, SM22a expression was significantly reduced in the β-GP group (*P* < 0.01), which increased significantly in the 3-PO group (*P* < 0.01, Figure 5A, 5D). In addition, 3-PO significantly decreased the number of β-GP-induced calcified nodules by 56.43% *(P* < 0.01, Figure 4E, 4F). These results indicate that 3-PO suppresses the osteogenic transition of VSMC calcification induced by β-GP and subsequent VC by inhibiting PFKFB3.

### Effect of κ-OR stimulation on VSMC calcification induced by lactate

To further visualize the effect of κ-OR stimulation against VSMC calcification by lactate, we investigated changes in the expression of osteogenic differentiation proteins influenced by U50,488H in the presence of lactate. In our study, VSMCs were cultured with or without lactate for five days. Results showed that the expression of RUNX2 and BMP2 was significantly increased in the lactate-treated group (*P* < 0.01, Figure 5A–5C), while SM22a expression decreased significantly (*P* < 0.01) (Figure 5A, 5D). After treatment with U50,488H, the expression of RUNX2 and BMP2 was significantly decreased (*P* < 0.05), while the expression of SM22a was significantly increased (*P* < 0.01). These effects of U50,488H were reversed by nor-BNI treatment (*P* < 0.05). Besides, lactate treatment significantly increased calcium deposition by approximately 106.36%, as assessed by Alizarin red staining after 14 days. However, U50,488H significantly relieved calcium deposition, decreased by 83.08%, while the effect of U50,488H was abolished by nor-BNI (*P* < 0.01, Figure 5E, 5F). Furthermore, ALP activity was significantly increased by up to 104.52 % with lactate-induced calcification in VSMCs for five days, which was inhibited by up to 37.72% treatment with U50,488H *(P* < 0.05, Figure 5G). This effect of U50,488H was abolished by nor-BNI treatment (*P* < 0.01, Figure 5G). These data reveal that κ-OR stimulation suppresses lactate-induced VSMC calcification.

### Effect of κ-OR stimulation on mineralization of aortic rings

**Figure 6:**
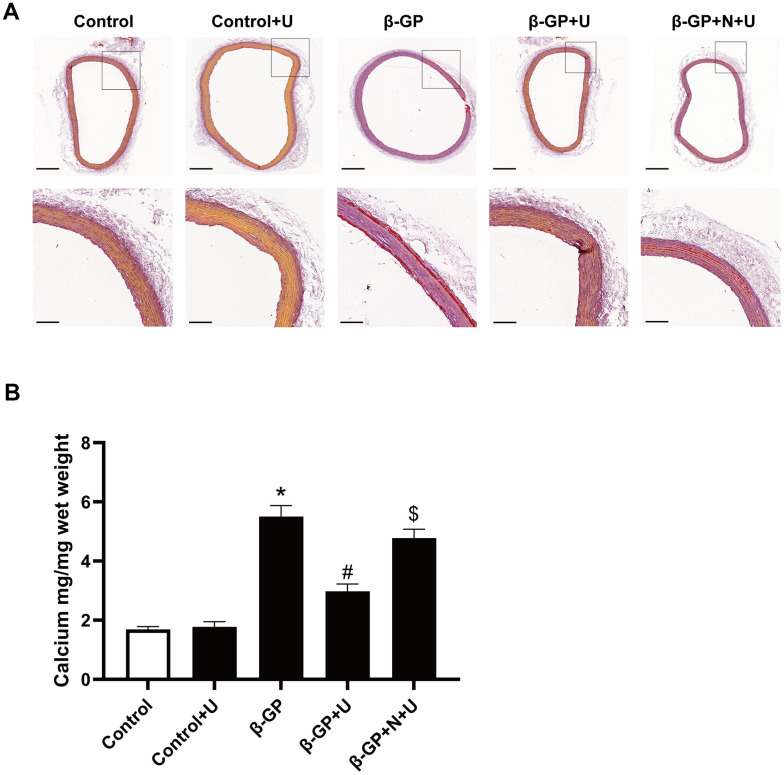
**Effects of κ-OR stimulation on β-GP-induced mineralization in aortic rings from rats.** (**A**) Representative Alizarin red-stained sections of aortic rings from rats; Scale bar = 500 μm (above panel) and 100 μm (below panel). (**B**) Quantification of mineralization in the aortic rings using the O-cresol phthalein complex one assay. Aortic rings from rats were incubated in control medium (Con; serum-free DMEM), phosphate medium (β-GP; serum-free DMEM + 10 mmol/L β-GP), phosphate medium + U50,488H (40 mmol/L), or phosphate medium + nor-BNI (80 mmol/L) + U50,488H (40 mmol/L) for ten days. U, U50,488H; β-GP, β-Glycerophosphate disodium salt pentahydrate; N, nor-BNI; Data are shown as mean ± SEM and were analyzed using one-way ANOVA tests. n = 4 in each group. ^*^P < 0.05 versus the control group, ^#^*P* < 0.05 versus the β-GP group, ^$^*P* < 0.05 versus the β-GP+U group.

To further confirm the effect of κ-OR stimulation on the mineralization of aortic rings, we examined whether U50,488H suppressed the mineralization of vessels induced by β-GP. In this experiment, we developed an *ex vivo* aortic calcification model based on the rat aortic ring, which was stained with Alizarin red after β-GP treatment for four weeks. Mineralization was assessed using histology, and calcium levels were measured using the o-cresol phthalein complex assay. Our results showed a significant increase by up to 3.12-fold mineralization after β-GP treatment (*P* < 0.05). U50,488H effectively prevented this change, which was decreased by up to 44.21% (*P* < 0.05), and the effect of U50,488H was abolished by nor-BNI (*P* < 0.05, Figure 6A, 6B). These data suggest that κ-OR stimulation reduces β-GP-induced VC in rat aortic rings.

## DISCUSSION

VC represents the pathological heterotopic accumulation of calcium and phosphate in the intimal and medial layers of the arteries, characterized by transdifferentiation of VSMCs from a contractile phenotype into an osteochondrogenic one, while matrix remolding events co-occur [34, 35]. Intracellular calcium overload triggers superoxide metabolism disruption, subsequently inducing high aerobic glycolysis [17, 36]. Accumulation of glycolytic products may promote VC, but the mechanisms remain elusive [18], indicating that interference with glycolysis may influence VC.

A previous study’s findings suggested that U50,488H significantly prevented an isoproterenol-induced increase in myocardial lipid peroxidation and depletion of myocardial antioxidants (glutathione, superoxide dismutase, and catalase) [37]. The enhanced intracellular Ca^2+^ transient and L-type Ca^2+^ currents elicited by isoprenaline in cardiomyocytes were significantly inhibited by U50,488H, both of which provide evidence that U50,488H reduces myocardial calcium influx and oxidative stress [31]. However, our previous study demonstrated that administration of U50,488H significantly decreased mean pulmonary arterial pressure and right ventricular hypertrophy, showing promise in attenuating vascular remodeling [38]. We also found that U50,488H interacted with the calcium-sensing receptor (CaSR) in the pulmonary artery and inhibited pulmonary hypertension and vascular remodeling through the CaSR/MAPK signaling pathway [28]. The above studies show that κ-OR protect the cardiovascular system by inhibiting intracellular calcium signals after activation, suggesting that κ-OR stimulation may exert a regulatory effect on cardiovascular tissue calcification.

Regarding cellular bioenergetics of VSMCs, we exposed rat aortic VSMCs to a phosphate donor, β-glycerophosphate, mimicking VC during hyperphosphatemia [39]. In a pro-calcifying environment, we found that calcium phosphate precipitation and ALP activity significantly elevated and increased levels of osteochondrogenic proteins, such as Runx2 and BMP. U50,488H treatment effectively inhibited the osteogenic transformation of VSMCs induced by β-GP. Morphological experiments support this effect of U50,488H. Alizarin red staining revealed that U50,488H inhibited the expression of Runx2 in a concentration-dependent manner. This U50,488H effect on calcification was blocked by nor-BNI, a selective κ-opioid receptor antagonist, indicating that κ-opioid receptors may be involved in VC regulation. This effect of U50,488H on calcification has also been demonstrated in studies with rat *ex vivo* aortic calcification rings.

Subsequently, we observed the expression levels of PFKFB3 and lactate to investigate whether the effect of U50,488H-induced attenuation of calcification was related to glycolysis. As critical kinases in glycolysis, PFKFBs synthesize and degrade PFK-1, a rate-limiting enzyme that catalyzes the conversion of fructose-6-phosphate (F6P) to fructose-1,6-bisphosphate (F1,6P2), and PFKFB3 is the only inducible isoform mainly expressed in vascular cells [13]. Upregulated PFKFB3 reportedly mediates collagen synthesis and proliferation of pulmonary artery SMCs, contributing to vascular remodeling in pulmonary arterial hypertension [40]. Our study detected higher levels of PFKFB3, lactate, and LDH activity in a pro-calcifying environment induced by β-GP. Conversely, PFKFB3 and glycolytic products were notably reduced after treatment with U50,488H. Moreover, PFKFB3 inhibition by the chemical inhibitor 3-PO attenuated VSMC calcification, which indicated that PFKFB3 might alleviate VC upon κ-OR stimulation.

As a set of membrane receptors, κ-ORs have been identified prominently in the vascular system [41]. PFKFB3, a key enzyme in glycolysis, is widely present in the nucleus of VSMCs [42]. Therefore, what seems puzzling is the interaction between the κ-ORs and PFKFB3 and their regulatory mechanism. Previous studies have shown that U50,488H has a protective effect on pulmonary vessels of rats exposed to chronic hypoxia [43]. In multiple diseases, hypoxia acutely influences PFKFB3 expression [13, 40, 44]. Then, we further confirmed that U50,488H repressed the PFKFB3 expression by upregulating the PHD2. We further observed the association between ectopic expression of nuclear PFKFB3 when κ-OR is activated. The present study indicated that U50,488H significantly inhibited PFKFB3 expression in β-GP-treated VSMCs; this effect was blocked by nor-BNI. These results suggest that PFKFB3 inhibition efficiently suppressed osteogenic differentiation and VSMC calcification induced by β-GP.

Previous studies have suggested κ-OR stimulation protects against hypoxic pulmonary hypertension (HPH) by inhibiting PASMCs autophagy [30]. In a hypoxic environment, VSMCs undergo phenotypic changes that can lead to vascular dysfunction, such as vascular inflammation and calcification [45]. Hypoxia contributes to VC by inducing osteochondrogenic differentiation of VSMC in a HIF-1a–dependent and mitochondria-derived reactive oxygen species–dependent manner [21]. Of the three PHD isoforms, PHD2 appears to be the primary HIF-1a hydroxylase based on genetically engineered mice and cell culture studies [46]. In the present study, we have shown that the PHD2 regulated PFKFB3 expression in VSMCs during calcification. However, the detailed mechanisms and inter-molecular interactions warrant further investigation.

Lactate is the final product of glycolysis and is directly regulated by PFKFB3 [13, 47]. Previous studies have suggested that lactate accelerates calcification in VSMCs, and our study confirmed similar results [17]. Our study found that U50,488H inhibited osteogenic differentiation of VSMCs in the high lactic environment. These results were also confirmed by Alizarin red staining and the ALP activity assay. These experimental results indicate that U50,488H effectively inhibits the lactate-induced osteogenic transformation of VSMCs.

Our study also has some limitations. The increase in κ-OR stimulation may affect the glucose enzyme metabolism, especially HIF-1α, an important molecule in VSMC calcification [24]. A follow-up study will investigate the relationship between κ-OR and key enzymes of glucose metabolism. Another limitation of our study lies in the physiological experiments; the number of experiments about functional effects is small, ultimately irrelevant from a translational point of view. Further studies will be required to provide direct evidence for glucose enzyme metabolism and VC studies *in vivo*.

In summary, we have demonstrated that activating κ-OR by its selective agonist U50,488H attenuates osteochondrogenic transdifferentiation prominently induced by β-GP, which may be mediated by inhibition of PFKFB3 expression and lactate. This study provides an experimental, theoretical basis to understand the pathogenesis and prevention of VC.

## MATERIALS AND METHODS

### Animals

Adult Sprague-Dawley male rats (age 4-6 weeks, weighing 150-200 g) were supplied by the Animal Center of the Fourth Military Medical University. Rats were housed with free access to food and water under pathogen-free conditions using a 12 h light and 12 h dark cycle. All animal experiments were performed in accordance with institutional guidelines and abided for the Care and Use of Laboratory Animals published by the U.S. National Institutes of Health, NIH Publication No. 85–23 (revised 1996 and approved by the University Ethics Committee of the Fourth Military Medical University).

### Cell culture and identification

Primary rat VSMCs were isolated according to previously published methods [48]. After attachment, the VSMCs (6 × 10^5^ cells per well) were cultured in Dulbecco's modified Eagle's medium (DMEM), containing streptomycin and penicillin, 2 mmol/L L-glutamine, and 12% FBS (GIBCO, USA) (see Supplementary Figure 1 for experimental details). VSMCs were identified by immunofluorescence, following subculture from 6-9 generations, for further experiments (please refer to Supplementary Figure 2 for experimental details).

VSMCs were randomly divided into ten groups. (1) Control group: normal culture in a normoxic incubator with 12% FBS; (2) Con+U group: U50,488H (Sigma, USA, 40 mm/L), a selective κ-opioid receptor agonist, was administered on the foundation of the control group; (3) β-GP group: β-glycerol (Sigma-Aldrich, USA, 10 mmol/L) was administered for ten days to establish a calcification model according to the literature [49]; (4) β-GP+U group: U50,488H (40 mm/L) was administered 10 min before treatment with β-glycerol; (5) β-GP+N+U group: nor-binaltorphimine, MCE, USA, 5 mm/L), a selective κ-opioid receptor antagonist, was administered 20 min before treatment with β-GP. After the administration of nor-BNI for 10 min, U50,488H was administered; (6) β-GP+U+IOX2 group: IOX2, MCE, USA, 5 mm/L), a selective PHD2 antagonist, was administered 15 min before treatment with β-GP. After the administration of IOX2 for 10 min, U50,488H was administered; (7) Control+3-PO group: 3-PO, a new type of small molecule PFKFB3 isoenzyme inhibitor, was administered on the foundation of the control group; (8) β-GP+3-PO group: 3-PO was administered 20 min before treatment with β-GP; (9) Control+Lactate group: Lactate (Solarbio, Beijing, China, 10 mmol/L) was administered on the foundation of the control group; (10) Control+Lactate+U group: U50,488H (40 mm/L) was administered 10 min before treatment with lactate; (11) Control+Lactate+N+U group: nor-BNI (5 mm/L) was administered 10 min before treatment with U50,488H, and then the cells were treated with β-glycerol (10 mmol/L) 10 min later.

### Immunofluorescence (IF)

Primary rat VSMCs cells (6 × 10^5^ cells per well) were seeded in six-well plates with 12 % FBS at room temperature for 24 h. After washing in phosphate-buffered saline (PBS), cells were fixed for 30 min at 37° C in 4% paraformaldehyde, blocked with 3% bovine serum albumin (BSA) for 1 h, and incubated overnight at 4° C in PBS containing anti-rabbit α-SMA antibody (CST19245, 1:200). Subsequently, cells were washed three times with PBS and incubated for 45 min at 37° C with anti-rabbit secondary antibody Alexa Fluor 488 Donkey anti-Rabbit IgG (H+L) (34206ES60, 1:200). The images were acquired using an immunofluorescence microscope (Nikon, Tokyo, Japan) with ×40 magnification.

### Measurement of the calcium content and ALP activity

The calcium content of VSMCs was determined using a Calcium Assay kit (Nanjing Jian Cheng Institute, Jiangsu, China) and normalized to the total protein content using the bicinchoninic acid (BCA) protein assay kit (Beyotime, Shanghai, China).

For ALP activity determination, the VSMCs pretreated with β-GP were solubilized with RIPA (Beyotime Shanghai, China). After centrifugation, the supernatants were examined with the ALP activity kit (Nanjing Jian Cheng Institute, Jiangsu, China) and normalized to the total protein content.

### Alizarin red staining

For Alizarin red staining, the VSMCs were fixed with 4% paraformaldehyde (Sigma) for 15 min at room temperature, rinsed twice with PBS, and stained with 0.1% Alizarin red (Solarbio, Beijing, China) for 30 min. The excess reagent was removed and rinsed twice with ddH_2_O.

Aortic specimens were fixed in 4% paraformaldehyde, embedded in paraffin, and cut into 4 μm thick sections. Sections were deparaffinized, stained with 2% Alizarin red for 10 min, and rinsed with PBS. Thereafter, sections were immersed in anhydrous acetone solution for 40 s, anhydrous acetone-xylene (volume ratio = 1:1) solution for 15 s, and xylene for 1 min twice. The calcium phosphate salts were visualized by red staining.

Calcium nodules were observed and photographed using an inverted microscope (Nikon, Japan). The quantification of the calcium deposits was assessed by measuring the optical density (OD) at 405 nm.

### Lactate and LDH measurements

VSMCs (5 × 10^5^) were seeded in 6-well plates. After incubation (72 h) at room temperature, cell culture media were collected to determine the lactate content, measured using a lactate assay kit (Nanjing Jian Cheng Institute, Jiangsu, China). Lactate production was determined using a linear range of standard lactate concentrations according to the manufacturer’s instructions, and the colorimetric method was used to measure the absorbance at 530 nm.

To test for released lactate viability, lactate dehydrogenase (LDH) levels were measured in VSMCs using an LDH assay kit (Nanjing Jian Cheng Institute, Jiangsu, China) and a standard (Roche, Indianapolis, IN, USA).

### Western blotting analysis

VSMCs were lysed according to the manufacturer’s instructions, and the protein concentration was measured using a BCA protein quantification kit (Pierce Biotechnology). Runt-related transcription factor 2 (RUNX2) (12556; 1:1000) was obtained from Cell Signaling Technology (Danvers, MA, USA). Anti-bone morphogenetic protein 2 (BMP-2) (ab14933), anti-TAGLN/Transgelin (SM22a, 1:1000), anti-PFKFB3 (ab181861), and anti-GAPDH (ab8245) were purchased from Abcam (Cambridge, MA, USA) (1:1000). Subsequently, 15-25 μg of protein was separated via 10% SDS-PAGE and then transferred onto nitrocellulose membranes. The membranes were blocked with 5% BSA for 1 h. Thereafter, membranes were incubated with primary antibodies overnight at 4° C, followed by incubation with anti-rabbit or anti-mouse IgG conjugated with horseradish peroxidase at 37° C for 1 h. The blots were detected using electrochemiluminescence (ECL), and the results were quantified using Quantity One software (1.8.0 version).

### Laser confocal microscopy assays

VSMCs cells (1 × 10^5^ cells per well) were seeded in 15 mm-diameter culture dishes and cultured in DMEM with 12% FBS at 37° C for 72 h. Cells were washed with PBS, fixed in 4 % paraformaldehyde, and blocked with 3% BSA for 1 h. Cells were then incubated with goat anti-rabbit PFKFB3 (ab181861, 1:200) at 4° C overnight. Subsequently, cells were incubated with Alexa Fluor 488 Donkey anti-Rabbit IgG (H+L) (34206ES60, 1:200) at room temperature for 45 min and imaged using a confocal laser scanning microscope (Olympus FV 1000, Olympus Corporation, Tokyo, Japan) at 488 nm to observe PFKFB3 nuclear translocation. Images were obtained every 10 s, and the relative fluorescence intensity was measured using Image J software.

### Rat model of calcification

A rat model of thoracic aorta calcification was developed as previously reported [50]. A total of 20 SD male rats, aged 6-8 weeks (150-200 g), were used in this experiment. After an adaptation period of one week, rats received vitamin D_3_ (300,000 IU/kg in arachis oil, intramuscularly) and nicotine (25 mg/kg in 5 mL peanut oil, intragastrically) dissolved in peanut oil. The rats were administered a nicotine/peanut oil mixture 8 h later. Four weeks later, extensive calcification appeared in the medial vascular area.

### *Ex vivo* rat aortic ring assay

Calcification model rats were anesthetized by intraperitoneal injection of pentobarbital sodium (60 mg/kg). The thoracic aortas were carefully dissected from the perivascular fat and connective tissues, and collected in Kerb’s solution (0–4° C). The adventitia was carefully removed, and the intimal surface was scraped to remove endothelial cells. Next, thoracic aortas were cut into 5-6 mm vascular rings and cultured *in vitro* based on a previously published method [51]. Rings were randomly divided into five groups. (1) Control group: aortic rings were incubated in serum-free DMEM (GIBCO, USA). The medium was changed every 48–72 h for ten days; (2) Con+U group: U50,488H (Sigma, USA,40 mm/L) was administered on the foundation of the control group; (3) β-GP group: β-glycerol (MCE, USA,10 mm/L) was administered for ten days; (4) β-GP+U group: U50,488H (40 mm/L) was administered 10 min before treatment with β-glycerol; (5) β-GP+N+U group: nor-BNI (nor-binaltorphimine, MCE, USA, 5 mm/L), a selective κ-opioid receptor antagonist, was administered 20 min before treatment with β-GP. After the administration of nor-BNI for 10 min, U50,488H was administered, and aortic rings were collected, fixed, and embedded in paraffin. Some sections were then stained with Alizarin red for calcification. Calcium was extracted from other rings using hydrochloric acid (0.6 mmol/L) for 1 h. The concentration of calcium was determined by the o-cresol phthalein complex ketone method and normalized to weight.

### Statistical analysis

Unless otherwise stated, the data were expressed as the mean ± SEM and analyzed using GraphPad Prism 8.0 (GraphPad Software, San Diego, CA, USA). Differences among groups were compared using one-way ANOVA followed by a pairwise comparison using the SNK-q method. Statistical significance was set at *P* < 0.05.

## Supplementary Material

Supplementary Figures
