# Supplementary material for: κ-opioid receptor stimulation alleviates rat vascular smooth muscle cell calcification via PFKFB3-lactate signaling
Source: Aging (Albany NY). 2021 May 20;13(10):14355–71. doi: 10.18632/aging.203050 (PMC8202865; doi:10.18632/aging.203050)
Supplement: Supplementary Figures [file aging-13-203050-s001.pdf]

SUPPLEMENTARY FIGURES

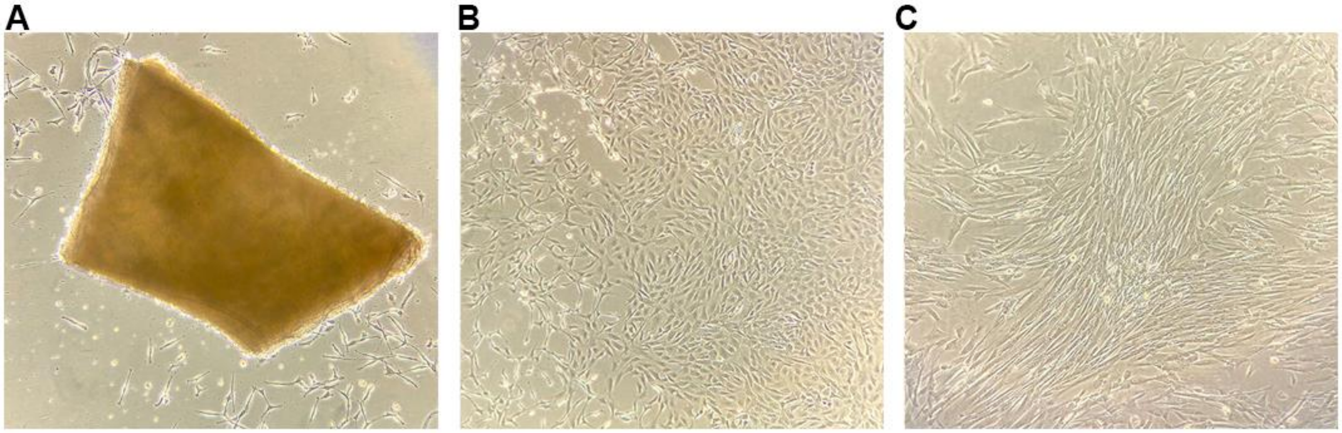

Supplementary Figure 1. Evaluation of VSMCs morphology in rat thoracic aorta. The isolated rat thoracic aorta vascular tissue block was inoculated and cultured. After 2-4 days, the cells could be seen crawling out of the edge. After 6-10 days of culture, the cells grew rapidly, spreading in a spindle shape, and fused into pieces. Passage after reaching 80% density, the characteristic "peak-valley" phenomenon of smooth muscle cells can be seen during the growth process. (**A**) VSMCs crawled out of the tissue block. (**B**) VSMCs fusion after 6-10 days. Quantitative analysis of RUNX2 expression. (**C**) Peak-valley phenomenon. (magnification: ×40).

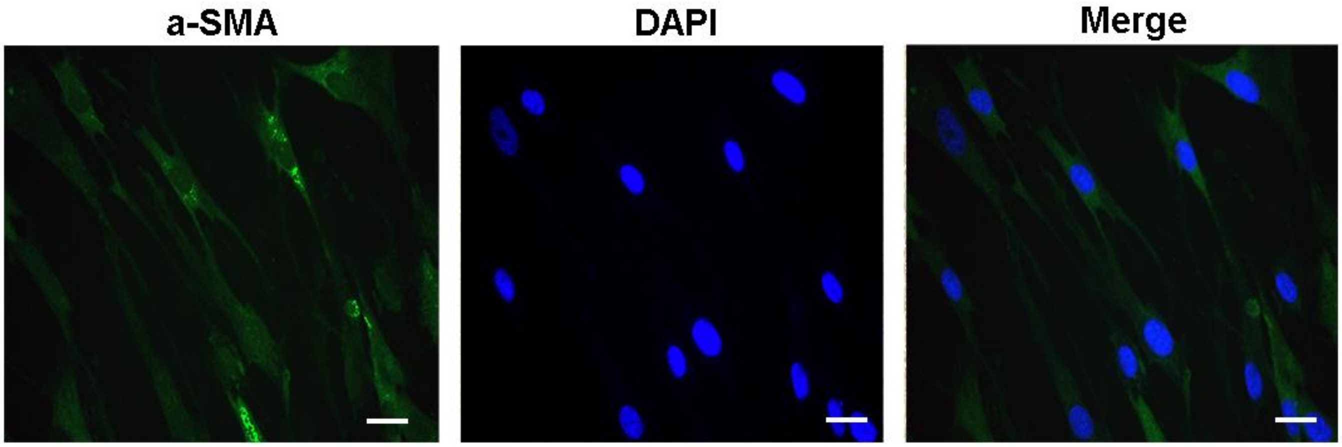

Supplementary Figure 2. Identification of vascular smooth muscle cells (VSMCs). Expression of α-SMA in VSMCs was determined using immunofluorescence (magnification: ×400). VSMCs at passage 6 were used for smooth muscle-specific protein (α-SMA) immunofluorescence staining and the result was obtained from a confocal microscopy. A large number of actin filaments parallel to the long axis (green fluorescence) in the cytoplasm was observed and regarded as α-SMA-positive cells. The nuclei were stained with DAPI. The percentage of α-SMA positive cells was more than 95%. Scale bar = 20μm.
